# Adaptability of the Sense of Agency in Healthy Young Adults in Sensorimotor Tasks for a Short Term

**DOI:** 10.3390/bs13020132

**Published:** 2023-02-05

**Authors:** Mizuho Mishima, Kazuki Hayashida, Yoshiki Fukasaku, Rento Ogata, Kazuki Ohsawa, Ken Iwai, Wen Wen, Shu Morioka

**Affiliations:** 1 Department of Physical Therapy, Faculty of Health Science, Kio University, Nara 635-0832, Japan; 2 Neurorehabilitation Research Center, Kio University, Nara 635-0832, Japan; 3 Department of Neurorehabilitation, Graduate School of Health Sciences, Kio University, Nara 635-0832, Japan; 4 Research Into Artifacts, Center for Engineering, Department of Precision Engineering, The University of Tokyo, Tokyo 113-8656, Japan

**Keywords:** sense of agency, adaptability, incongruence

## Abstract

Sense of agency (SoA) refers to the subjective feeling of controlling one’s own actions and sensory feedback. The SoA occurs when the predicted feedback matches the actual sensory feedback and is responsible for maintaining behavioral comfort. However, sensorimotor deficits because of illness cause incongruence between prediction and feedback, so the patient loses comfort during actions. Discomfort with actions associated with incongruence may continue robustly (i.e., “not” adaptable) throughout life because of the aftereffects of the disease. However, it is unclear how the SoA modulates when incongruency is experienced, even for a short term. The purpose of this study was to investigate the adaptability of the SoA in healthy participants in sensorimotor tasks for a short term. Participants were divided into congruent and incongruent exposure groups. The experimental task of manipulating the ratio of the self-control of a PC cursor was used to measure the SoA before and after exposure to congruent or incongruent stimuli. The results showed no significant differences between the groups before and after exposure for a short term. The finding that the SoA was not adaptable may assist in guiding the direction of future studies on how to correct incongruence.

## 1. Introduction

In everyday life, healthy people can move their bodies and manipulate objects as intended while rarely making mistakes in their actions. What makes this seamless behavior possible is an internal model of sensory predictions based on motor commands [1,2,3,4]. The subjective feeling that occurs when this prediction matches the actual sensory feedback is called the sense of agency (SoA) [5,6]. SoA is a hierarchical system with a lower sensorimotor level consisting of sensory predictions and sensory feedback and a higher cognitive level consisting of beliefs and context. A mechanism has been proposed in which the SoA is determined by the cognitive level when the sensorimotor level is ambiguous [7]. This would be analogous to a blind person relying more heavily on hearing to navigate the world. Furthermore, the SoA is responsible for maintaining behavioral comfort and is known to differ developmentally from children to the elderly [8,9,10,11]. This suggests that the SoA can be flexible enough to adapt to changes in the body and mind over a long period of time. However, once an individual becomes ill, abnormalities in the SoA occur, including those in neurological disease [12,13,14], Parkinson’s disease [15], schizophrenia [16], and autism spectrum disorders [17,18]. Patients have an incongruence between their predictions and sensory feedback each time an action is performed. This incongruence, and thus the reduced SoA, can lead to discomfort with actions, such as a sense of heaviness and dysesthesia [19,20].

Neurological diseases such as stroke are likely to result in residual motor deficits [21,22]. Specifically, discomfort with actions associated with incongruence may continue robustly (i.e., “not” adaptable) throughout life. Although the robustness of visual perception is well known, in the SoA it is based on motor perception and is unknown. Furthermore, individual differences in psychological states, such as depressive symptoms, schizotypy, and sensory sensitivity, may affect adaptability based on motor perception, but their relationship is also unknown. 

Surprisingly, it is unclear how SoA modulates when experiencing incongruence even in sensorimotor tasks for a short term. Recently, Wen et al. developed an experimental task to detect changes in SoA by experimentally manipulating the self-control ratio of a PC cursor [23,24]. This experiment manipulates the touchpad (prediction) and monitors cursor movement (feedback) to 100% self-control (i.e., fully congruent), partial self-control, or 0% self-control (i.e., fully incongruent). This experimental task may allow us to examine the adaptability of the SoA during a brief experience with incongruence between prediction and feedback.

In summary, it is unclear whether the incongruence associated with sensorimotor deficits continues throughout life (i.e., is not adaptable) or whether it adapts to motor deficits and updates its internal model to reduce incongruence (i.e., adaptability) in sensorimotor tasks for a short term. In addition, the relationship between the psychological traits that influence adaptability based on motor perception is unclear. Therefore, this study aimed to investigate the adaptability of the SoA and the relationship between the SoA and psychological factors in healthy participants. To achieve this purpose, we examined whether the SoA changes between the pre- and post-exposure phases, in which participants experience incongruence for a brief period of time, using the dot task. If the SoA is not adaptable, then it should remain unchanged before and after the exposure phase. If the SoA is adaptable even for the short term, it may remain adaptable in the long term, and the development of procedures to correct this incongruence will be required in the future. Alternatively, if the SoA is adaptable, it is important to investigate the mechanism of how quickly it adapts to incongruence. In summary, we believe that this study will assist future research in this area.

## 2. Materials and Methods

### 2.1. Participants

Thirty-three healthy participants (mean age = 21.5, SD = 0.9) participated in this study. We randomly divided the participants into a Congruence group (16 participants comprising eight females) and an Incongruence group (17 participants comprising eight females). The sample size was chosen based on the power calculation using G*Power 3.1.9.2 with repeated-measures within-between interaction: effect size = 0.4, α = 0.05, power = 0.95, number of groups = 2, number of measurements = 2, corr among rep measures = 0.5, and non-sphericity correction ε = 1. The total required sample size was twenty-four participants. Additionally, the sample size was taken from a similar motor control task study (one group consisted of 12 or 17 participants) [25]. All participants reported the visual, hearing, verbal, and finger functions required for the experiment. The Kio University Ethics Committee approved the study procedures (approval number: R4-17) and the experiments were conducted per the Declaration of Helsinki.

### 2.2. Materials

The task and measuring system were created using MATLAB and Psychtoolbox (MathWorks, Natick, MA, USA). A personal computer (FUJITSU LIFEBOOK UH90/C3, Minato City, Japan) with a 13.3-inch display was used for all tasks and to record the data.

### 2.3. Procedure

#### 2.3.1. Experimental Task

**Figure 1:**
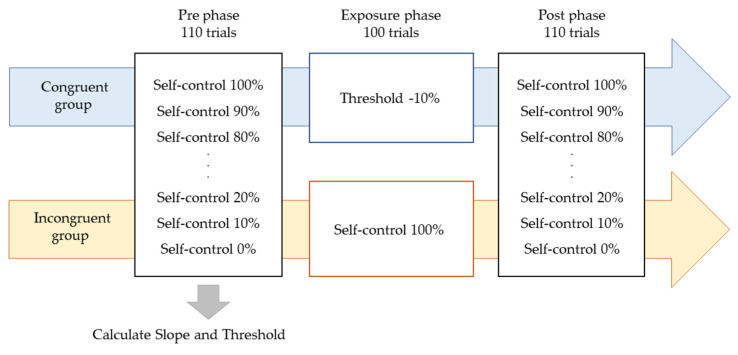
Experimental procedure. The experiment consisted of three phases: the Pre phase, Exposure phase, and Post phase. In the Pre and Post phases for both the Congruent and Incongruent groups, participants performed 10 trials each at 10% intervals, starting at 0% and ending at 100% self-control conditions at random. Participants in the Incongruent group in the exposure phase performed 100 trials of the dot manipulation task at a value 10% lower than the individual’s threshold calculated in the Pre phase. The Congruent group was exposed to 100 trials under the 100% condition.

**Figure 2:**
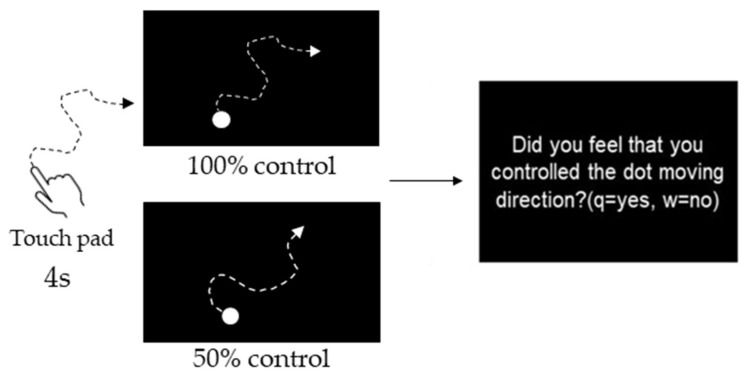
Experimental task. Participants were instructed to manipulate a dot on a PC screen using a touchpad and to manipulate the dot freely within 4 s. They were evaluated on whether they felt in control. Figure 2 shows an example of 100% or 50% control.

Participants were instructed to manipulate a dot (40-pixel) on a PC screen using a touchpad and to manipulate the dot freely within 4 s. The experiment consisted of three phases: the Pre phase, Exposure phase, and Post phase (Figure 1). In the Pre and Post phases for both the Congruent and Incongruent groups, participants performed 10 trials each at 10% intervals, starting at 0% and ending at 100% self-control conditions at random (i.e., 110 trials). After each trial, participants were evaluated on whether they felt in control. Figure 2 shows an example of 100% or 50% control. In this task, participants were instructed to answer “Yes” if they felt that they were in control of the movement, even if they felt uncomfortable with the movement of the dots [24]. The threshold values were calculated using logistic curve regression based on the responses obtained in the Pre phase (see below). Participants in the Incongruent group in the exposure phase performed 100 trials of the dot manipulation task at a value 10% lower than the individual’s threshold calculated in the Pre phase (i.e., a situation that generated non-self-movement awareness). For example, if the threshold was 50%, the participants were exposed to a 40% condition. The Congruent group was exposed to 100 trials under the 100% condition (Figure 2). These tasks were created using MATLAB (MathWorks, Natick, MA, USA), PsychToolbox, and R2016a. Participants completed two practice tasks of 100%, 60%, and 0%, and performed the experimental tasks only after they fully understood the tasks. It took approximately 30 min to complete all experimental tasks.

#### 2.3.2. Psychological Assessment 

Regarding the Beck Depression Inventory (BDI)-II [26], higher scores indicated more severe depressive states. Furthermore, regarding the Schizotypal Personality Questionnaire (SPQ) [27], higher scores indicated more severe schizotypy states. To measure the severity of sensory sensitivity states, we used the Highly Sensitive Person Scale (HSPS)-J19 [28], and higher scores indicated more severe sensory sensitivity. 

### 2.4. Data Analysis 

**Figure 3:**
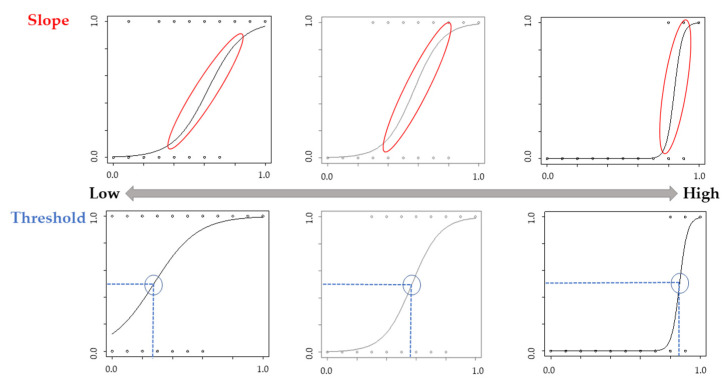
Image of the slope and threshold. A high positive slope indicates that the participants were judged as having their own movement, whereas a low slope indicates that participants ambiguously judged the movement as their own. A high threshold value indicates that they did not judge the movement as their own unless it reflected more of their own movement, whereas a low threshold value indicates that the movement was judged as one’s own, even if it did not reflect one’s own movement significantly.

Logistic regression curves for each participant were used to calculate the “slope” of the curve and the points of subjective equality (i.e., “threshold”) when participants gave 50% of the trials “Yes” or “No” answers [29]. A high positive slope indicates that the participants were judged as having their own movement, whereas a low slope indicates that participants ambiguously judged the movement as their own. A high threshold value indicates that they did not judge the movement as their own unless it reflected more of their own movement, whereas a low threshold value indicates that the movement was judged as one’s own, even if it did not reflect one’s own movement significantly (Figure 3). These values were calculated using R version 4.2.1. Analysis using the Kolmogorov–Smirnov test showed that the threshold and slope data were normally distributed. We analyzed the threshold and slope using a two-way analysis of variance (ANOVA) with group (Congruence and Incongruence) as the within-subject factor and phase (Pre and Post) as the between-subject factor. Data above two standard deviations (SD) were excluded. A Mann–Whitney U test was used to compare the groups in the psychological assessment. Additionally, the relationship between each psychological assessment and the change in values ((Pre-Post)/Pre) of the threshold or slope was analyzed using Spearman’s rank correlation coefficient. Statistical significance was set at *p* < 0.05. HAD ver. 16 was used as the analysis software [30]. 

Given a small sample containing relatively scant information about the population parameters, Bayesian estimates are more heavily weighted toward the prior. It is the influence of the prior distribution that helps stabilize and anchor Bayesian parameters in the presence of small samples. Therefore, in an additional analysis, considering the slope and threshold, we analyzed using Bayesian estimation with Markov chain Monte Carlo (MCMC) methods, which are not directly affected by sample size. Rstan ver. 2.26.13 was used as the analysis software.

## 3. Results

### 3.1. Slope and Threshold

**Figure 4:**
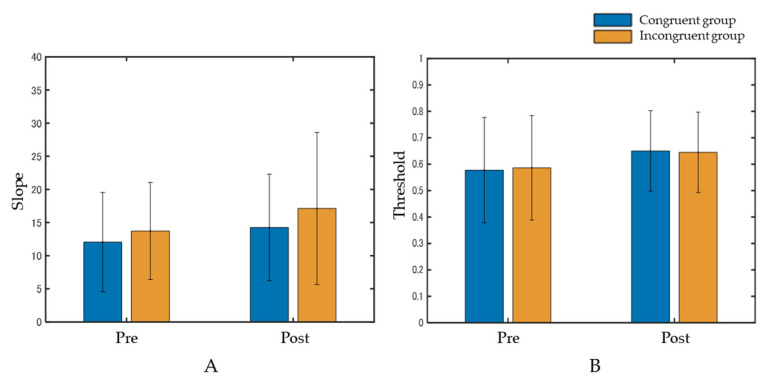
Results of the slope (**A**) and the threshold (**B**). Data represent means ± standard deviation. Both the interaction and main effects were not significant.

At the slope, the interaction between the group and phase was not significant (F(1, 58) = 0.063, *p* = 0.80, η2p = 0.001). The main effects were also not significant in the group (F(1, 58) = 0.76, *p* = 0.39, η2p = 0.01) and phase (F(1, 58) = 1.59, *p* = 0.21, η2p = 0.03) (Figure 4A). Regarding the threshold, the interaction between the group and phase was not significant (F(1, 58) = 0.023, *p* = 0.88, η2p = 0.0004). The main effects were also not significant in the group (F(1, 58) = 0.001, *p* = 0.96, η2p = 0.00002) or phase (F(1, 58) = 2.16, *p* = 0.15, η2p = 0.04) (Figure 4B).

### 3.2. Psychological Assessment

There were no significant differences between the groups on any psychological scale (Table 1). 

**Figure 5:**
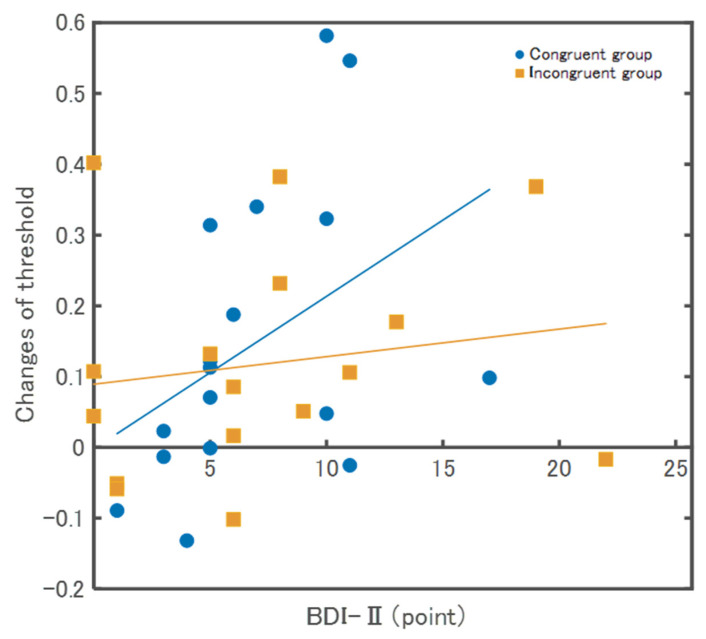
Results of correlation between changes of threshold and BDI-Ⅱ. The Congruent group showed a significant positive correlation (ρ = 0.52, *p* = 0.03), whereas, in the Incongruent group, there was no significant correlation (ρ = 0.13, *p* = 0.62).

The relationships between the threshold and the psychological scales are described below. The BDI-II in the Congruent group showed a significant positive correlation (ρ = 0.52, *p* = 0.03), whereas, in the Incongruent group, there was no significant correlation (ρ = 0.13, *p* = 0.62) (Figure 5). In the SPQ, there were no significant correlations between the Congruent (ρ = 0.18, *p* = 0.47) and Incongruent groups (ρ = 0.01, *p* = 0.97). In the total HSPS-J19 score, there were no significant correlations between the Congruent group (ρ = 0.09, *p* = 0.74) and the Incongruent group (ρ = −0.23, *p* = 0.39). For factor 1 (low sensory threshold) of HSPS-J19, there were no significant correlations between the Congruent (ρ = 0.17, *p* = 0.66) and Incongruent groups (ρ = −0.41, *p* = 0.12). For factor 2 (ease of excitation) of HSPS-J19, there were no significant correlations between the Congruent group (ρ = 0.06, *p* = 0.81) and Incongruent group (ρ = −0.23, *p* = 0.39). For factor 3 (aesthetic sensitivity) of HSPS-J19, there were no significant correlations between the Congruent (ρ = 0.34, *p* = 0.19) and incongruent groups (ρ = 0.15, *p* = 0.59). 

The relationships between slope and all psychological scales were not significantly correlated in either group. In the BDI-II, there were no significant correlations between the Congruent group (ρ = 0.22, *p* = 0.43) and the Incongruent group (ρ = 0.07, *p* = 0.80). In the SPQ, there were no significant correlations between the Congruent (ρ = 0.06, *p* = 0.82) and Incongruent groups (ρ = −0.004, *p* = 0.99). In the total score of HSPS-J19, there were no significant correlations between the Congruent (ρ = 0.21, *p* = 0.45) and Incongruent groups (ρ = 0.16, *p* = 0.62). For factor 1 (low sensory threshold) of HSPS-J19, there were no significant correlations between the Congruent (ρ = 0.10, *p* = 0.72) and Incongruent groups (ρ = 0.02, *p* = 0.95). For factor 2 (ease of excitation) of HSPS-J19, there were no significant correlations between the Congruent group (ρ = 0.24, *p* = 0.38) and Incongruent group (ρ = 0.07, *p* = 0.79). For factor 3 (aesthetic sensitivity) of HSPS-J19, there were no significant correlations between the Congruent (ρ = −0.22, *p* = 0.42) and Incongruent groups (ρ = 0.31, *p* = 0.24).

In the additional analysis, a total of 4000 MCMC samples were used for estimation, with 4 chains, 2000 iterations, and 1000 warm-ups and Rhat < 1.1 for all parameters. At the slope, there was no interaction between the group and phase (95% credible interval (−1.95, 1.36)), no main effect of group (95% credible interval (−3.73, 1.83)), and no main effect of phase (95% credible interval (−0.17, 3.02)). At the threshold, there was no interaction between the group and phase (95% credible interval (−0.02, 0.01)) and no main effect of the group (95% credible interval (−0.06, 0.07)), whereas there was a main effect of the phase (95% credible interval (0.02, 0.05)). These Bayesian results mostly supported the results of the frequentist statistics.

## 4. Discussion

This study investigated the adaptability of the SoA by experimentally exposing congruent or incongruent sensory predictions and actual feedback for a short term. There was no interaction between pre- and post-exposure thresholds or slopes in the Congruent and Incongruent groups. The interaction in the additional analysis also showed a relatively high likelihood of the null hypothesis. Thus, these results indicate that the SoA may not be adaptable. Hence, the SoA targeted by this experimental task should be primarily generated at the sensorimotor level of processing. Only in the Congruent group was the change in threshold pre- and post-exposure positively correlated with depression, suggesting a relationship with psychological factors. The following is a discussion of the results:

The non-adaptability of the SoA suggests that it is difficult to accept incongruence. In a previous study of stroke patients using a task to control the cursor of a monitor, it was shown that misattribution of the cursor (i.e., a factor of reduced SoA) was associated with less frequent use of the upper limb with sensorimotor deficits [14]. If the SoA is not adaptable, the patient may choose not to use the paralyzed limb to avoid incongruent discomfort. We hypothesize that this process may contribute to learned non-use [31]. If this hypothesis were correct, intervention procedures would need to be developed in future studies to modify incongruence and enhance the SoA. Sensorimotor deficits cause incongruence owing to the input of abnormal sensory feedback. Therefore, medical and rehabilitation interventions are necessary to treat sensorimotor deficits. Depending on the pathology, the possibility that the prediction mechanism or internal model itself is impaired should also be considered [6]. It is necessary to identify where the pathology lies in the predictive mechanisms and sensory feedback and to modify the incongruence according to the pathological mechanisms.

The change values of threshold and depression were positively correlated only in the Congruent group. The effect in the changed threshold values was partially consistent with the results showing a high likelihood of an alternative hypothesis in the additional analysis phase. Several previous studies have reported that the SoA is reduced by stronger depression because participants’ movements cannot be attributed to themselves [32,33]. In contrast, we suggest that the experience of congruence with depressive tendencies may improve the SoA. However, repeated incongruent experiences did not change the SoA. Although rehabilitation is necessary to improve sensorimotor deficits, patients often experience depression and low motivation [34], and a reduced SoA may be involved in these psychological states [35]. Therefore, from the perspective of the SoA, an errorless learning task without incongruence should be an effective choice for the rehabilitation of patients with depression and low motivation.

To confirm the non-adaptability of the SoA, several possible limitations of this study should be considered. First, the results were based on our sensorimotor task, which was limited to the sensorimotor level with no cognitive-level cues. If cognitive-level procedures had been set up, the SoA could have been adapted flexibly [7]. Patients with impaired sensorimotor levels may rely on cognitive-level cues. This can offer a new alternative intervention to enhance the availability of other agency cues or use technology to generate new cues for agency. Second, when this experimental task was not in the 100% self-control condition, the movement of the dots was not patterned and the error could not be predicted. Motor tasks, such as Imamizu’s (2000), have been shown to have implicitly patterned errors, and the internal model was updated as the task was repeated [36]. Thus, research on the changes in the SoA after experiencing patterned errors is needed. Third, in the present experimental task, participants were only required to freely manipulate the dot, which was not purposeful. We reported that the SoA was flexibly enhanced in experiments with a purposeful perceptual motor learning task, where participants were required to update their internal model [25,37,38]. If they experienced a purposeful motor task, the SoA may have changed, and thus should be tested in future studies. Fourth, in the present experimental task, the participants responded to just two options (yes or no). In the recent study, the participants were able to respond to three options (yes, no, or “partial”) [24]. Since our participants knew that they were partly in control, most of the time, it was difficult to ascertain if participants were genuinely considering whether they felt that they controlled the dot. Finally, the maximum exposure time in this experiment was 10 min. An examination of the effects of prolonged exposure is necessary for patients exposed to lifelong incongruence. After a long period of incongruence, the contexts and beliefs of the body may be altered, and how the SoA is modulated by the interaction between the sensorimotor and cognitive levels should be examined in the future.

## Figures and Tables

**Table 1:** Group comparison of psychological assessment.

	Congruent Group		Incongruent Group		U	*p*-Values
	Median	IQR	Median	IQR		
BDI-II ^1^	5	5–10	6.0	1–9.5	134.0	0.96
SPQ ^2^	20	11–28	17.5	13.75–25.5	122.0	0.63
HSPS ^3^						
-Total score	80	72–92	82.0	74.75–95.75	115.5	0.47
-Factor 1	29	25–34	28.5	23–33.75	124.0	0.68
-Factor 2	37	29–43	35.0	33–38.75	138.0	0.93
-Factor 3	18	17–20	18.5	18–21.25	112.5	0.4

^1^ BDI-II = Beck Depression Inventory-II; ^2^ SPQ = Schizotypal Personality Questionnaire; ^3^ HSPS = Highly Sensitive Person Scale.

## Data Availability

The data presented in this study are available in the Supplementary Materials here.

## Supplementary Materials

The following supporting information can be downloaded from https://www.mdpi.com/article/10.3390/bs13020132/s1, Table S1: Dataset.
